# A Bibliometric Analysis on Nonpoint Source Pollution: Current Status, Development, and Future

**DOI:** 10.3390/ijerph18157723

**Published:** 2021-07-21

**Authors:** Ping Lei, Ram Kumar Shrestha, Bing Zhu, Suju Han, Hongbin Yang, Shaojun Tan, Jiupai Ni, Deti Xie

**Affiliations:** 1 College of Resources and Environment, Southwest University, Chongqing 400715, China; tracy7909@163.com (P.L.); ram.shrestha@lac.tu.edu.np (R.K.S.); yanghobin@163.com (H.Y.); tsj2015@126.com (S.T.); 2 School of Urban and Rural Planing and Constrution, Mianyang Teachers’ College, Mianyang 621000, China; peipei1975@126.com (B.Z.); hansu76@126.com (S.H.); 3 Lamjung Campus, Institute of Agriculture and Animal Science, Tribhuvan University, Kirtipur 33603, Nepal

**Keywords:** nonpoint source pollution, bibliometric analysis, geographic distribution, keyword analysis

## Abstract

Nonpoint source pollution (NPS) has become the leading factor of global water quality problems, attracting great attention from governments and researchers in various countries. Based on this situation, understanding the current research status of NPS can help guide future research. However, most of the current reviews only describe the research status of some specific aspects but fail to quantify the research hotspots and development trends on the whole, which limits the overall understanding of NPS. In this paper, bibliometrics was used to study the current status, hotspots, and frontiers of NPS research during 1991–2015, and the future research development was predicted. Over the past 15 years, there has been a remarkable growth trend in publication output, and the participation of countries/territories has also increased. *Journal of Environmental Quality*, *Journal of Hydrology*, and *Total Environmental Science* were the top three journals. Sharpley AN and Arnold JG from the USA were the most productive authors with the best quality articles. The major author clusters and research regions are located in North America and Europe, followed by East Asia. The United States dominates this research field, with the largest number of independent and collaborative articles. Chinese authors gained more attention through international cooperation. Keyword analysis confirmed that water quality and nutrients were the main concerns of NPS pollution research, which mainly involved a number of research topics, such as pollutant emission reduction research and the evaluation and simulation of pollutants’ migration and their transformation under different situations, while pesticides were less of a concern, which suggests that the abuse of pesticides has come under control. Meanwhile, SWAT was the dominating model in the last decade partly because it satisfied the growing needs of watershed-scale management.

## 1. Introduction

Water, essential for human existence and development, is a non-renewable resource [1]. The deterioration of water quality is not only one of the most serious environmental problems in the world but also one of the major social and economic issues [2]. As we well know, after the point sources have been effectively controlled, nonpoint source pollution (NPS) mainly caused by agricultural activities (such as livestock and poultry breeding, field irrigation, excessive use of pesticides and fertilizers, etc.) becomes the single most important source threatening the utilization of water resources [3,4,5]. NPS pollutants may remain active in the environment for a relatively long period and could cause chronic effects on aquatic ecosystems as well as human health [3,6]. International research on agricultural NPS began with statistics and analysis of observational data and proposed corresponding policies and agricultural management measures.

With the development of hydrological models and crop growth models, researchers used these models to simulate the agricultural nonpoint source process, discussed the pollution load under different management methods, found the key source areas of NPS, and discussed related control measures [7,8]. Therefore, NPS research is of great importance to prevent and reduce NPS risk and to improve the capacities of controlling pollution. Despite tremendous explorations and practice, governments and scholars still have not found a suitable solution to address the NPS issue worldwide. To solve this problem, scholars began to adopt interdisciplinary research methods, especially the introduction of GIS spatial analysis technology. Its powerful spatial analysis, spatial positioning, and other functions are conducive to the exploration of the spatial and temporal differences of agricultural NPS risk and to carrying out the prevention and control of agricultural NPS [9]. Therefore, it is particularly important to refine and analyze the existing literature related to NPS, including summarizing the main research content of the literature and research difficulties and predicting future research directions.

Bibliometrics theory believes that the amount of scientific literature and scientific knowledge usually has a synchronous growth trend, and the growth law also has similarities. The changes in the number and structure of scientific and technological literature and the laws of mutual citation reflect the development characteristics of the corresponding scientific and technological fields [10]. Bibliometrics is an effective tool that applies mathematics and statistics to literature and other communication media [11,12]. Scientific literature objectively records the development of each discipline or knowledge field. An analysis of the development trend of disciplines based on bibliometrics enables one to systematically summarize the research status and hot issues in this field and evaluate the scientific research capabilities and output of research institutions. This is of great significance to grasp the discipline development trend, track the latest progress, select innovative topics, and improve the efficiency of scientific research [13,14,15]. At present, relying on large-scale analytical databases and bibliometric analysis methods, scholars have carried out bibliometric research in many fields and achieved certain scientific achievements [11,14,15,16,17,18]. However, only a few comprehensive bibliometric studies have been performed so far on the global NPS research literature. Zhang et al. [19] mainly studied and analyzed international nonpoint source trends in the number of publication outputs, subject categories and major journals, geographic and institutional distribution of publications, and keyword analysis during the period 1975–2010. They choose “Non point source*” or “Nonpoint source*” (including “non point source”, “non point sources”, “non-point source”, “non-point sources”, “nonpoint source”, “nonpoint sources”, and “nonpoint-source”, etc.) as a search term to search for all publications that contained these words in the title, abstract, and keywords. However, they might have omitted some articles such as those using “diffuse* pollut*” as a noun phrase which can represent nonpoint source pollution.

In this study, CiteSpace III and HistCite12.3 software were used to analyze the current research status, research hotspots, and research frontiers of NPS from multiple perspectives, so as to provide references and suggestions for future NPS research.

## 2. Materials and Methods

We built our bibliometric database using publications on NPS from the Science Citation Index Expanded (SCIE) and Social Sciences Citation Index (SSCI), which are regarded as the most frequently used and comprehensive literature databases for bibliometric studies [20]. An accurate search query is the first and most important step in the retrieval process. Liu [21] considered that omission or misuse of non-alphabetic characters in a search filter may lead to inaccurate results and wrong conclusions. Therefore, we employed nearly all phrases representing NPS (“non point source*”, “nonpoint source*”, “nonpoint pollut*”, “non point pollut*”, “diffuse* pollut*”, “diffuse* source*”, “surface source *pollut*”, etc.) as search terms to obtain all articles that contain these words in titles, abstracts, or keywords from 1991 to 2015. The data was retrieved on 13 April 2016. The downloaded content includes “full records and cited references”, and the data format was “plain text” format, which was then imported into CiteSpace III and HistCite 12.3 software for analysis [22]. Then, we subsequently combined all the records and deleted duplicated records.

Collaboration type was determined by the address of each author, where “single-country” was assigned for papers with authors from only one country or institute, and “Internationally collaborated” were assigned for papers with authors from more than one country [23].

Pathon, Google Earth, and ArcGIS software were used to analyze the geographical distribution of the authors. First, authors’ affiliations were extracted through CiteSpace III, and the number of articles in each country/region were calculated. Second, the authors’ location coordinates were obtained by using Python-based Geoprocessing Library (GeCopy). Third, their reverse geocoding was performed using the Google Maps API. Finally, ArcGIS software was used to draw the global geographic distribution zoning based on the author’s location.

The total citation score (TC) on Web of Science includes all the cited records in WoS database. However, not all citations are related to the target study. Some articles are cited in non-target research; we call these non-target citations. Total local citation score (TLCS) is the number of times a paper is cited by other papers in the local collection. Total global citation score (TGCS) is the number of times a paper is included in the collection cited in WoS [24]. In this study, local citation score (LCS) refers to the index that one paper was cited in the NPS database. LCS removed the non-NPS research citations, which we did not need, and only showed the cited papers within the “NPS Database”. In this study, TGCS and TLCS were acquired by using HistCite12.3 software. Additionally, we gathered the 5-year impact factors of journals and subject categories in our bibliographic database from the 2015 ISI Journal Citation Report (JCR).

If the frequency of a term has significantly increased in a short time, we can infer that this term represents a developing trend. How to detect the mutation of the term in a short time is an important task for us to carry out for the trend analysis in the field of science. The burst detection algorithm can be used for the detection of the sudden increase of scientific interest, whereby theme mutations are analyzed quantitatively and qualitatively. The burst detection algorithm is proposed by Kleinberg in 2003 [25]. To obtain a more intuitive representation of the evolution of NPS research, we use CiteSpace III software first to extract noun phrases from the downloaded data records, identifying bursts of emerging terms.

All publications were analyzed as follows: (a) Document types and publishing languages; (b) Research output indicators; (c) Subject categories and major journals; (d) Productive countries and internationally collaborated; (e) Geographic distribution dynamics of institute publication activity; (f) Analysis of keywords and hot issues.

## 3. Results and Discussion

### 3.1. Document Types and Publishing Languages

We analyzed the distribution of document types recognized by WoS and found 16 document types in 6337 NPS-related publications. The largest number of articles was 6009, accounting for 94.80% of the total number of published articles. Proceeding papers (774; 12.2%) and reviews (208; 3.3%) also occurred in a significant portion of the publications accessed. Other less significant document types included meeting abstracts (45), editorial material (36), news items (17), book chapter (12), corrections (6), notes (5), discussions (4), correction addition (2), reprints (2), letters (1), book review (1), database review (1), and bibliography (1).

As journal articles often have absolute predominance in all document types, articles are usually chosen for analysis in bibliometric research using the Web of Science database [26]. In this study, journal articles were the central issue, and all other types of publications were discarded for further analysis. As for the publishing language, 5936 journal articles were written in English, followed by French (26), German (20), Portuguese (15), Spanish (4), Polish (4), Lithuanian (1), Croatian (1), Danish (1), and Chinese (1), which were minor publication languages in NPS Pollution research. In conclusion, English is the leading scientific language, and most ISI-indexed journals were published in English.

### 3.2. Research Output Indicators

The publications devoted to NPS during 1991–2015 are shown in Table 1. It was observed that the annual number of articles, authors per paper, cited references, and average citations per article increased considerably. The number of total articles (TA) increased nearly sevenfold from 66 in 1991 to 429 in 2015. The average number of references (NR/TA) increased from 19.3 in 1991 to 48.8 in 2015, suggesting that the knowledge base of NPS research is constantly expanding. Another indicator of substantial development in this field was the collaboration index, which was defined as the average number of signatories on a single paper [27]. In this study, the average authors per article (NA/TA) grew from 2.4 in 1991 to 4.0 in 2015, indicating that studying NPS is a more collaborative field of research. In addition, the average number of citations per article (TC/TA) during the study period reached 23.6, indicating that this field has received high attention. Overall, these indicators of research output have shown steady growth, and collaboration in the field of NPS research has also been strengthened.

### 3.3. Web of Science Categories and Major Journals

**Figure 1:**
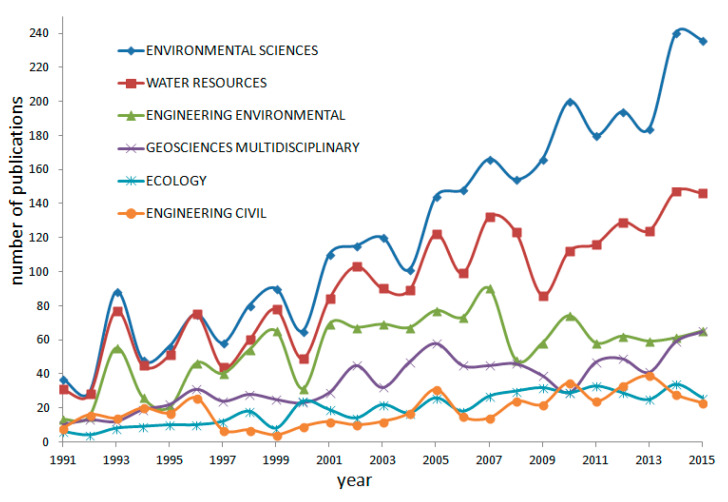
The growth trends of the top 6 Web of Science subject categories.

NPS-related research covered 138 Web of Science subject categories which were published in the Science Citation Index Expanded (SCIE) and Social Sciences Citation Index (SSCI). The number of subject categories increased from 22 in 1991 to 42 in 2000, and to 57 in 2015, reaching a peak of 61 in 2014. The six most productive categories of NPS research are shown in Figure 1. Environmental sciences (3081; 51.3% of) and water resources (2237; 37.2%) were far ahead of the other subject categories. The other four subject categories were engineering environmental (1359; 22.6%), geosciences multidisciplinary (883; 14.7%), ecology (490; 8.2%), and engineering civil (467; 7.8%). During the study period, NPS research has increased in the categories of geosciences multidisciplinary, especially environmental sciences and water resources, while it has fluctuated slightly in engineering environmental, ecology, and engineering civil.

The number of papers published in the field of environmental science and water resources has always taken the leading position and maintained a strong momentum of development, which also confirmed that nonpoint source pollution is actually a problem of water environment pollution.

A total of 6009 articles have been published in a wide range of 778 journals in 138 subject categories in WoS. This manifested the breadth of publication distribution as well as the extensive interest in NPS research from various perspectives. In terms of the most relevant journals researching in the NPS field, Table 2 showed the top 10 most productive journals with the total number of articles, total local citation score (TLCS), total global citation score (TGCS), average citation frequency of an article in the local collection, 5-year impact factors (IF), and h-indexes of individual journals. The results showed that a total of 1856 NPS research papers were published in 10 core journals, accounting for 31% of the total published articles, which means these journals are most interested in research related to NPS, and researchers of this field also believe that these journals are the most effective channels for communicating research results in the field of NPS. In terms of the number of articles covered, the *Journal of Water Science and Technology* ranked first, followed by the *Journal of the American Water Resources Association*, and *Science of the Total Environment*. *Journal of Environmental Quality*, *Journal of Hydrology*, and *Science of the Total Environment* were on the front-ranking in ALCS, GCS, and IF-5 which indicated the highest strengths, showing their superiority and great academic influence in NPS research. In addition, *Environmental Science and Technology* ranked first in IF-5 and H-index but ranked 8th in ALCS, suggesting that the journal’s academic influence is very high but not widely in NPS.

### 3.4. Author Performance

We conducted an author productivity analysis, and the 20 most productive authors are listed in Table 3. These authors appeared to be research pioneers in fields related to NPS. Srinivasan R from the United States Department of Agricultural Research Service (USDA ARS) contributed the most articles (37), followed by Shen ZY from Beijing Normal University (34), Arnold JG from USDA ARS (31), and Sharpley AN from University Arkansas (30). The number of articles they published as the first author or the corresponding author (FCA) was also counted. The FCA of Shen ZY ranked first (26), and Kim LH from Korea University ranked second, indicating that they are both prolific writers.

In addition to article productivity (NA and FCA), the academic impacts of the authors were also shown with four indices: total citations (TC), citation per publication (CPP), H_i_ (an impact factor developed by Hirsch in 2005 that incorporates both quantity of publications and quality citations of a researcher’s scientific outputs), and H_m_ (a modified H_i_). Considering that our H-index statistical results are often the same rank, we use H_m_ to optimize results. In fact, once the H_i_ of scholars in the same discipline is the same, scholars’ academic impacts will be unable to sort. To overcome this defect of the H_i_, we revised H_i_. The H_m_ is represented as shown below:

H_m_ = H_i_ + H_i_/TC

Here, H_i_ is the Hirsch’s index, and TC is the total citations. The greater the difference between H_m_ and H_i_, the smaller the influence of the scholars in the field; the smaller the difference, the greater the influence.

Generally, the 20 authors were from three regions: North America, Europe, and Asia. North America and Europe accounted for 10 and 6 of the top 20, respectively. Furthermore, 6 out of the 10 authors in North America and 4 out of the 6 authors in Europe had a very high academic impact index, ranking top 10 in TC, CPP, or H_m_ (only Walter MT ranked 11 in H_m_). Besides North America and Europe, China also had three authors on the list. Two of them were ranked top 10 in FCA but not in the top 10 in the academic impact index (TC, CPP, and H_m_). This showed that North America and Europe have taken the lead in NPS over the past 25 years.

Considering the fact that TC_FCA_ (total FCA citations) has more far-reaching academic effects than TC, we also use TC_FCA_ and FCA_CPP_ (citation per FCA publication) to evaluate the authors’ academic impact. Arnold JG ranked first in TC_FCA_ and FCA_CPP_, and Sharpley AN ranked second in TC_FCA_ and FCA_CPP_, which indicated that Sharpley AN and Arnold JG had more high-quality articles in NPS in the past 25 years. Jarvie HP from the Centre for Ecology and Hydrology had only 22 articles but a high H-index of 18. Considering that older articles are likely to have higher citations, we used 5 years as a fixed analysis window. We find Sharpley AN ranked 3 in TC and 1 in CPP but ranked 10 in TC_FCA_ and 9 in FCA_CPP_. Meanwhile, Shen ZY ranked 10 in TC and 18 in CPP but ranked 1 in TC_FCA_ and 4 in FCA_CPP_. This shows that in the last five years, Sharpley AN’s team still has a high influence; his personal influence, however, significantly decreased, and Shen ZY’s team has gained increasing academic and personal influence.

### 3.5. Productive Countries and Internationally Collaborated

Table 4 shows the top 20 countries and regions in the world that produced the most articles on NPS from 1991 to 2015. The USA and China are the two most productive countries, with 2832 articles and 666 articles, respectively. However, in single-country publishing, the USA and Britain have 2436 publications and 264 publications, respectively, with the highest citation frequency of 61,158 publications and 6681 publications, respectively. China has 519 single-country publications, but the TC is only 4935. Articles by authors from the USA and UK are more cited than articles by Chinese authors. It can be inferred that the academic level of Chinese authors in research on NPS is not as high as their American and UK counterparts. Chinese researchers should work harder to improve the quality of the literature. The Netherlands and Sweden have the highest citations per paper, which suggests that the articles published by authors from these two countries are of outstanding academic value.

In internationally collaborated research, the USA and China still produce the most articles, and the USA and China have the highest total citations, which confirms that Chinese authors gained higher attention through international cooperation. In terms of the proportion of international collaborative research in the total research, Sweden, the Netherlands, Denmark, and New Zealand (86.61%, 53.33%, 49.33% and 45.65%, respectively) were the most active. Authors from these countries seem to be more interested in international research, possibly because they have a good tradition of international cooperation in scientific research. It is necessary to point out that the Netherlands has both the highest single-country AV-TC and internationally collaborated AV-TC, which means that the authors of this country have a high scientific level.

### 3.6. Geographic Distribution Dynamics of Institute Publication Activity

**Figure 2:**
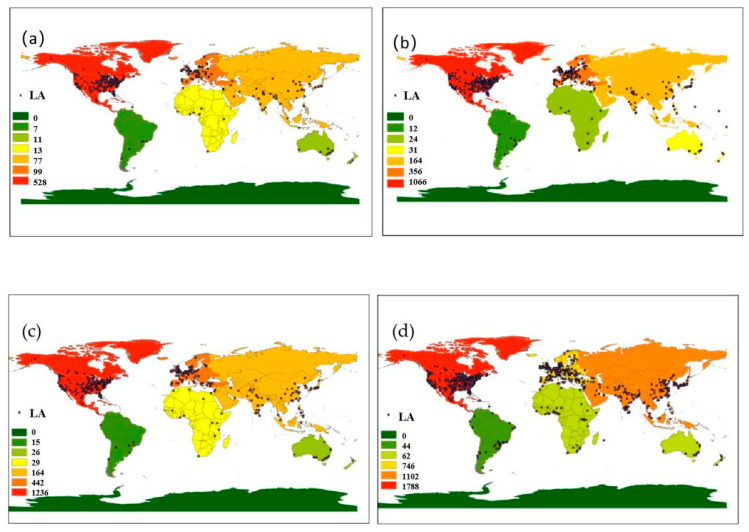
Global geographical distribution of authors based on institution location during (**a**) 1991 to 1997; (**b**) 1998 to 2003; (**c**) 2004 to 2009; and (**d**) 2010 to 2015. Color shades represent the number of articles, and the intensity of spots represents geographic distribution of authors. LA: Location of authors.

There were 14,886 authors in 6009 articles, and author affiliations were extracted to calculate the number of articles for each country/region. We produced a map (Figure 2) to show how global NPS research output has changed over the past 25 years.

From 1991 to 1997, the research on NPS was mainly concentrated in North America and Western Europe, and the United States was the only research hotspot. During this period, only 754 articles were published globally and just 45 were published in Asia. From 1998 to 2003, research in the field expanded to most continents in the world. However, in Asia, Oceania, Africa, and South America, there are still few institutions involved, and their publishing activities are generally low. From 2004 to 2009, the geographical expansion of NPS research was at its peak, with more institutions participating in the research. There were three hotspots in Asia: Beijing, Nanjing, and Seoul. Furthermore, the total number of articles increased from 951 in 1998–2003 to 1178 in 2004–2009. From 2010 to 2015, the number of participating institutions, and consequently the total number of articles published, increased rapidly, reaching 2306.

The major spatial clusters of authors were located in North America, Western Europe, and East Asia. There are relatively few authors from Africa, South America, Eastern Europe, West Asia, Central Asia, and Southeast Asia. The USA-based authors were mostly located on the east coast. The clusters of Western European authors were mainly distributed in the UK, Germany, and Italy, whereas the clusters of East Asian authors were mainly positioned in Eastern China and South Korea. The distribution of authors and countries increased significantly in the last 5 years of our study period. These authors also spread from developed to developing countries and from coastal to inland regions. Different from the scattered distribution of European and American research institutions, the geographical distribution of Asian research institutions is more concentrated, with seven participating institutions in Seoul and more than ten in Beijing. The research hotspots in the United States revealed the high activity of the USDA Agricultural Research Service (USDA ARS). Fort Collins, Colorado, was the hot spot for the first ten years of the study period, partly because it is the location of four research institutes of the USDA ARS that studied NPS pollution. Some other research hotspots (Madison, Wisconsin; Temple, Texas; Ames, Iowa; and University Park, Pennsylvania) also have one or two USDA ARS research institutes.

### 3.7. Analysis of Keywords and Hot Issues

Statistical analysis of author keywords can be used to identify the research direction and interest of researchers and to discover trends and frontiers in scientific research [26,28]. In this study, there are 1157 without any keywords, and there are 11,325 author keywords in the remaining 4852 articles. A total of 8713 (76.9%) author keywords only appeared in one article, 1203 (10.6%) appeared two articles, and 457 (4%) appeared in more than three articles. A majority of less frequently used keywords were associated with a lack of close correlation with NPS and a lack of continuity in the research. The number of keywords used more than 10 times was only 284 (2.5%), and these keywords were used in mainstream NPS-related research, which indicates that the mainstream research on NPS focused on a small area.

Table 5 shows the 30 most frequently used author keywords along with their rankings and percentages, which were calculated and ranked using 6-year intervals (except for the first period, which is a 7-year interval) to minimize year-to-year fluctuations. “nonpoint source pollution” and “Water quality” were the most frequently used keywords in NPS pollution articles during the study period, which shows that nonpoint source pollution has a great impact on water quality [5]. Apart from “nonpoint source pollution” and “Water quality”, “Phosphorus” and “Nitrogen” were the most frequently used words, confirming that agricultural nonpoint sources have become the main cause of global water quality deterioration [29]. As we all know, due to the increase in the use of phosphorus and nitrogen fertilizers and the drastic changes in land use patterns, the phosphorus and nitrogen pollution in receiving water bodies around the world has increased dramatically. [30,31,32,33]. Among the top 30 author keywords, “runoff”, “sediments”, “land use”, “Nutrient(s)”, “Watershed/River basin”, “Eutrophication”, and “agriculture” and were ranked 6th, 8th, 12th, 14th, 15th, 16th, and 25th, respectively.

Soil erosion is the main source of nonpoint source pollution, and rainfall and land use change are important factors influencing soil erosion [26,33]. Global climate and land use changes may severely affect soil erosion, leading to nonpoint source pollution, which in turn affects the water quality and safety of rivers and lakes [30].

Model is another hot keyword in NPS-related research. Many models can be used to simulate and evaluate NPS at a small watershed scale under complex agricultural management measures. To understand and evaluate the processes of pollution generation, transport, and transformation, modelling is undoubtedly a study tool which can simplify the complex natural process method. SWAT was the most commonly used model over the last 18 years. The rank of “swat” increased continuously from 180th in 1998–2003 to 26th in 2004–2009 and to 14th in 2010–2015. However, the frequency of pesticides as a keyword decreased from 1991 to 2015. This implies that agricultural abuse of pesticides has been brought under control.

Compared with 1991–1997, the rank of “water”, “groundwater”, “nitrate”, and “pesticide” was greatly improved throughout 1991–1997, which may be attributed to the implementation of the Environmental Quality Incentives Program (EQIP). EQIP was established in the “Federal Agriculture Promotion and Reform Act” of 1996. The primary goal of the project is to provide financial and technical support for producers to achieve the dual goals of improving agricultural production and environmental quality [34].

The keywords related to watershed-scale (including “watershed /River basin”, “River”, “catchment”, “Streams”, “Basins”, and “watershed management”) were consistent common keywords in recent years. This phenomenon implies that researchers in NPS pollution modeling have focused more on watershed-scale modeling than field-scale modeling in recent years [35].

In general, nonpoint source pollution mainly involves research topics such as the study of pollutant reduction and the assessment and simulation of the migration and transformation of pollutants under different conditions.

### 3.8. Research Trends

Table 6 shows the top 20 appearing terms in NPS research from 1991 to 2015. These high burst strength terms show high potential to guide future research and have a fundamental influence on future development. Among them, “China” had the highest burst strength (33.6163), followed by “SWAT” (16.708), and the lowest burst term is “galaxy” (5.1373). According to the character, frequency, and duration, the research status is divided into two types: “fading research frontier” and “the new research frontier”. In this paper, the frontier of fading research refers to the research topic reflected by the keywords, that is, the literature shows a trend of decline year by year.

The fading research frontier has 15 high-frequency highlighted keywords, such as “Groundwater“, “Herbicide”, “Geographic information system”, “atrazine”, “erosion”, “watershed management”, “pesticide”, “discriminant analysis”, “Midwestern united states”, “lake”, “corn”, ”nonpoint source”, “movement“, “AGNP”, and “Galaxy”. Scholars are less concerned with these keywords, which have become fade-type research frontiers to NPS. The latest research frontier refers to those keywords which had a climbing trend in the literature year by year. In this paper, six keywords have shown significantly high burst strengths in the past 5 years, such as “china”, “swat”, “climate change”, “impact”, and “area”. This will be the subject of emerging research in the field. China will be a research hot spot in NPS in the future. The SWAT model not only can simulate NPS load but also can identify NPS key areas and establish different management scenarios to evaluate the effectiveness of different optimal management practices. Therefore, SWAT has become a hot topic in NPS research in recent years.

**Figure 3:**
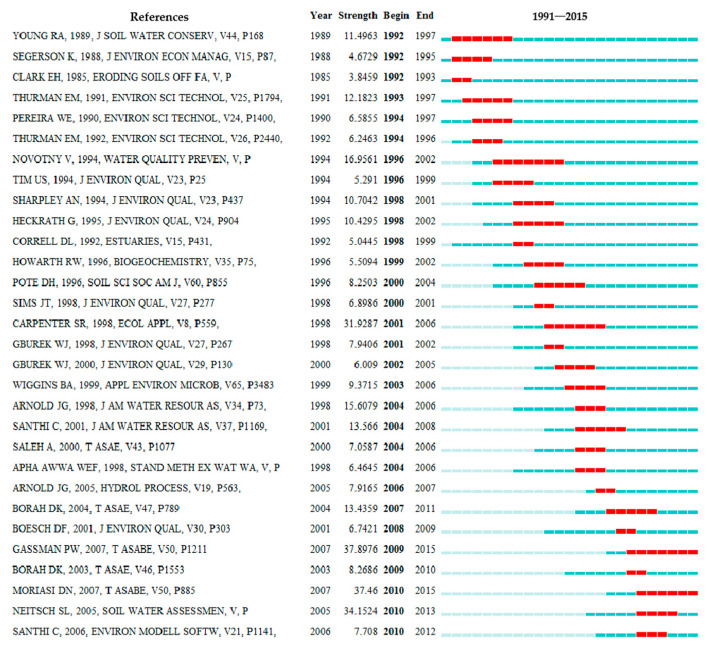
The top 30 references with the strongest citation bursts.

Combined with the evolution of nouns, this paper summarizes the current situation and hotspots of NPS research in the past 25 years through co-citation analysis (as shown in Figure 3). According to the time order, Figure 3 shows the 31 references with strong citation bursts. From 2009 to 2015, researchers focused on the NPS model, especially the SWAT model [36,37,38,39]. GASSMAN PW dissected the strengths and weaknesses of the SWAT model and recommended research needs for SWAT. BORAH DK reviewed the applicability of eleven watershed-scale hydrologic and nonpoint source pollution models. MORIASI DN applied comprehensive guidance to watershed model evaluation in terms of the accuracy of simulated data compared to measured flow and constituent values. SANTHI C evaluated the long-term impact of the implementation of water quality management plans (WQMPs) on NPS at the farm level and the watershed level using a modeling approach. SHEN ZY adopted the first-order error analysis (FOEA) method to analyze the effect of parameter uncertainty on SWAT model outputs under three types of land use, namely plantation, forest, and grassland.

To summarize, both high-frequency keyword analysis and sudden reference analysis point the popular research trend toward the nonpoint source pollution model, especially SWAT analysis. In SWAT analysis, it is an urgent problem to determine the recommended model evaluation technology (statistics and graphics), which is a hot research topic.

## 4. Conclusions

A systematic review of NPS pollution research from 1991 to 2015 based on bibliometric analysis revealed the worldwide research performance and the temporal evolutions of hot issues, which can be summarized as follows: (1) *Journal of Environmental Quality*, *Journal of Hydrology*, and *Total Environmental Science* have a huge academic influence in NPS research. (2) The main study area is distributed in North America and Europe with strong scientific research capabilities, followed by East Asia. The United States was the most active and influential country in NPS, possibly because the USDA ARS played a critical role. (3) In internationally collaborated research, the USA and China produced the most articles, and Chinese authors gained higher attention through international cooperation. (4) Water quality and nutrients were consistently the main concern of NPS pollution, while pesticides were less of a concern, suggesting that the agricultural abuse of pesticides has been brought under control. SWAT was the dominating model in the last decade partly because it satisfied the growing needs of watershed-scale management.

Overall, bibliometrics can identify research trends, hotspots, and frontiers of NPS, which will provide help for future research. The research may shed light on the comprehensive and systematic model of NPS research and help us better understand the progress in this field.

## Figures and Tables

**Table 1:** Scientific outputs on NPS from 1991 to 2015.

Year	TA	NA	NA/TA	NR	NR/TA	TC	TC/TA
1991	66	156	2.4	1276	19.3	1112	16.8
1992	66	165	2.5	1451	22.0	1485	22.5
1993	135	325	2.4	2885	21.4	3619	26.8
1994	106	268	2.5	3157	29.8	3827	36.1
1995	112	256	2.3	2625	23.4	2745	24.5
1996	141	388	2.8	3947	28.0	4775	33.9
1997	128	418	3.3	4258	33.3	5604	43.8
1998	175	520	3.0	5427	31.0	8910	50.9
1999	173	465	2.7	4736	27.4	5006	28.9
2000	149	509	3.4	4793	32.2	5493	36.9
2001	203	599	3.0	6460	31.8	6927	34.1
2002	227	699	3.1	7573	33.4	7171	31.6
2003	251	915	3.6	8140	32.4	7894	31.5
2004	237	746	3.1	7699	32.5	5786	24.4
2005	300	1013	3.4	10,188	34.0	7734	25.8
2006	288	1055	3.7	10,198	35.4	6696	23.3
2007	306	1067	3.5	11,277	36.9	6573	21.5
2008	324	1115	3.4	11,505	35.5	5706	17.6
2009	316	1137	3.6	12,763	40.4	5312	16.8
2010	361	1325	3.7	15,470	42.9	5297	14.7
2011	368	1431	3.9	15,986	43.4	4075	11.1
2012	369	1356	3.7	15,811	42.8	3012	8.2
2013	365	1406	3.9	16,911	46.3	1807	5.0
2014	414	1734	4.2	19,249	46.5	1278	3.1
2015	429	1728	4.0	20,926	48.8	330	0.8
Average	240.40	831.84	3.235	8988.4	34.03	4727	23.61
Total	6009	20,796	-	224,711	-	118,174	-

TA: total articles; NA: number of authors; NR: the number of references; TC: total citations received for published articles; NA/TA: average authors per article; NR/TA: average references per article; TC/TA: average citations per article.

**Table 2:** Top 10 most productive journals (1991–2015).

Journal	TA	TLCS	TGCS	ALCS(R)	IF-5	H_i_
*Water Science and Technology*	435	891	4092	2.05(9)	1.20	30
*Journal of the American Water Resources Association*	318	1333	7339	4.19(5)	2.23	37
*Science of the Total Environment*	197	817	5371	4.15(6)	4.32	39
*Journal of Environmental Quality*	175	1060	7704	6.06(1)	2.92	44
*Journal of Hydrology*	148	792	4321	5.35(2)	3.88	40
*Environmental Monitoring and Assessment*	143	164	1340	1.15(10)	1.92	18
*Environmental Science and Technology*	126	367	5594	2.91(8)	6.40	45
*Journal of Soil and Water Conservation*	121	571	2425	4.72(3)	2.35	26
*Water Research*	97	449	3474	4.63(4)	6.77	35
*Journal of Environmental Management*	96	392	2136	4.08(7)	4.05	28

TA: total articles; TLCS: total local citation score; TGCS: total global citation score; ALCS(R): rank of average citation frequency of an article in the local collection; IF-5: average impact factor of the past five years; H_i_: high citations index.

**Table 3:** The top 20 productive authors in NPS.

Author/Institute	TA	FCA(R)	TC(R)	CPP(R)	TC_FCA_(R)	FCA_CPP_(R)	Hi	Hm(R)	In 5-Year Window			
									TC(R)	CPP(R)	TC_FCA_(R)	FCA_CPP_(R)
Srinivasan R/USDA ARS	37	4(18)	3352(3)	90.6(3)	57(11)	6.9(18)	17	17.005(6)	392(2)	90.6(3)	0	0
Shen ZY/Beijing Normal Univ	34	26(1)	465(14)	13.7(18)	369(5)	10.9(10)	13	13.028(9)	81.4(10)	13.7(18)	223(1)	8.58(4)
Arnold JG/USDA ARS	31	4(18)	3672(2)	118.5(2)	005(1)	64.7(1)	20	20.005(2)	432.6(1)	118.5(2)	113(2)	28.3(1)
Sharpley AN/Arkansas Univ	30	13(4)	4539(1)	151.3(1)	792(2)	26.4(2)	21	21.005(1)	372.4(3)	151.3(1)	20(10)	1.5(9)
Mostaghimi S/Virginia Tech Univ	27	8(8)	519(12)	19.2(14)	39(13)	8.9(15)	14	14.027(8)	38.6(18)	19.2(14)	0	0
Huang GH/Regina Univ	26	13(4)	358(17)	13.8(17)	300(8)	11.5(9)	10	10.028(17)	46.2(15)	13.8(17)	112(3)	8.62(3)
Steenhuis TS/Cornell Univ	25	9(7)	756(8)	30.2(9)	63(10)	10.5(12)	17	17.022 (5)	92.4(7)	30.2(9)	0	0
Kim LH/Kongju National Univ	25	22(2)	367(16)	14.7(16)	343(7)	13.7(7)	8	8.022(19)	47.8(13)	14.7(16)	29(7)	1.3(10)
Neal C/Ctr Ecol and Hydrol Univ	24	7(13)	981(5)	40.9(7)	94(17)	8.1(17)	18	18.018(4)	94.8(6)	40.9(7)	0	0
Walter MT/Cornell Univ	23	8(8)	655(10)	28.5(10)	28(14)	9.9(13)	13	13.0198(11)	81.8(9)	28.5(10)	32(6)	4.0(6)
Chen L/Beijing Normal Univ	23	8(8)	162(20)	7(20)	22(20)	1.0(20)	7	7.043(20)	27.4(20)	7.0 (20)	22(9)	2.8(8)
Jarvie HP/Ctr Ecol and Hydrol Univ	22	8(8)	912(6)	41.5(6)	510(3)	23.2(3)	18	18.020(3)	97(5)	41.5(6)	1(13)	0.1(13)
Hao FH/Beijing Normal Univ	22	6(16)	248(19)	11.3(19)	04(19)	4.7(19)	10	10.040(15)	41.2(17)	11.3(19)	24(8)	4.0(6)
Behrendt H/Inst Freshwater Ecol and Inland Fisheries	22	7(13)	524(11)	23.8(11)	05(16)	9.3(14)	13	13.025(10)	47(14)	23.8(11)	0	0
Kleinman PJA/USDA ARS	21	4(18)	1024(4)	48.8(4)	56(12)	12.2(8)	13	13.013(13)	105.8(4)	48.8(4)	49(4)	12.3(2)
Engel BA/Purdue Univ	21	8(8)	453(15)	21.6(13)	84(18)	8.8(16)	11	11.024(14)	43.6(16)	21.6(13)	3(12)	0.4(12)
Chaubey I/Purdue Univ	21	11(6)	494(13)	23.5(12)	26(15)	10.8(11)	10	10.020(18)	68(12)	23.5(12)	48(5)	4.4(5)
Kronvang B/Aarhus Univ	20	7(13)	848(7)	42.4(5)	368(6)	18.4(5)	16	16.020(7)	87.8(8)	42.4(5)	0	0
Heathwaite AL/Lancaster Univ	20	5(17)	668(9)	33.4(8)	394(4)	19.7(4)	13	13.0195(12)	72.4(11)	33.4(8)	0	0
Tsihrintzis VA/ Athens Natl Tech Univ	19	17(3)	315(18)	16.6(15)	298(9)	15.7(6)	10	10.032 (16)	37.6(19)	16.6(15)	14(11)	0.8(11)

TA: total articles; FCA(R): rank of the first author or the corresponding author; CPP(R): rank of citation per publication; TC_FCA_(R): rank of total FCA citations; FCA_CPP_ (R): rank of citation per FCA publication; Hi: high citations index; H_m_: a Modified Hi.

**Table 4:** The top 20 countries in the world that publish the most articles on NPS.

Single-Country						Internationally Collaborated			
Country	TA	SP	TC	Av-TC	SP(%)	CP	TC	AV-TC	CP(%)
USA	2832	2436	61,158	25.11	86.02	396	6250	15.78	13.98
China	666	519	4935	9.51	77.93	147	2017	13.72	22.07
UK	495	264	6681	25.31	66.84	131	1715	13.09	33.16
Canada	301	184	2287	12.43	61.13	117	1502	12.84	38.87
Germany	293	178	2986	16.78	60.75	115	1227	10.67	39.25
France	210	136	2212	16.26	64.76	74	995	13.45	35.24
South Korea	198	159	880	5.53	80.30	39	262	6.72	19.70
Australia	175	113	2210	19.56	64.57	62	507	8.18	35.43
Japan	174	130	1738	13.37	74.71	44	707	16.07	25.29
Italy	147	98	2743	27.99	66.67	49	460	9.39	33.33
Spain	143	88	1822	20.70	61.54	55	477	8.67	38.46
Netherlands	135	63	2413	38.30	46.67	72	2020	28.06	53.33
Sweden	112	15	563	37.53	13.39	97	897	9.25	86.61
Scotland	103	61	757	12.41	59.22	42	265	6.31	40.78
India	97	78	1222	15.67	80.41	19	117	6.16	19.59
New Zealand	92	50	750	15.00	54.35	42	564	13.43	45.65
Greece	84	62	1125	18.15	73.81	22	204	9.27	26.19
Brazil	80	64	561	8.77	80.00	16	49	3.06	20.00
Taiwan	78	64	578	9.03	82.05	14	205	14.64	17.95
Denmark	75	38	270	33.42	50.67	37	614	16.59	49.33

TA: total articles; SP: single-country publication; TC: total citations; AV-TC: the average number of citations; SP (%): percentage of single-country publication; CP: internationally collaborated publication; CP (%): percentage of internationally collaborated publication.

**Table 5:** Thirty most frequent keywords in NPS articles.

Keywords	TP	91–97 R(%)	98–03 R(%)	04–09 R(%)	10–15 R(%)
nonpoint source pollution	2111(1)	1(30.5)	1(35)	1(34)	1(37.6)
Water quality	1157(2)	2(14.2)	2(19.6)	2(20.0)	2(20.2)
Phosphorus	782(3)	5(8.0)	4(11.4)	3(13.5)	3(15.1)
Nitrogen	755(4)	4(8.2)	3(11.9)	4(13.0)	4(14.0)
Model	710(5)	3(8.4)	6(9.9)	5(12.7)	5(13.22)
Runoff↑	641(6)	11(4.4)	5(10.7)	7(11.5)	6(12.0)
Pollution	639(7)	9(5.4)	6(9.9)	6(11.6)	7(11.9)
Sediments	585(8)	6(6.4)	9(8.2)	8(9.0)	9(11.3)
Management↑	515(9)	18(3.2)	16(5.4)	9(10.2)	8(11.6)
Soils	490(10)	10(5.0)	12(6.7)	10(8.2)	11(9.5)
Quality	454(11)	24(2.5)	10(7.0)	11(8.6)	12(8.8)
Land use	434(12)	28(1.9)	22(4.4)	12(8.5)	10(9.7)
Water	427(13)	8(5.6)	8(8.4)	13(7.4)	20(6.7)
Nutrient(s)	418(14)	12(3.8)	13(6.2)	14(7.2)	14(8.2)
Watershed/River basin↑	400(15)	20(2.9)	18(5.3)	16 (6.3)	13(8.7)
Eutrophication↓	364(16)	15(3.5)	11(6.9)	18(6.0)	21(6.5)
River	347(17)	21(2.8)	14(5.9)	20(5.4)	17(7.0)
Transport	341(18)	15(3.5)	18(5.3)	15(6.6)	23(5.9)
Catchment	318(19)	110(0.5)	32(2.7)	19(5.8)	16(7.7)
Nitrate	314(20)	13(3.7)	16(5.4)	16(6.3)	27(4.8)
Groundwater	302(21)	6(6.4)	15(5.6)	25(4.1)	28(4.5)
Systems	298(22)	8(5.6)	27(3.2)	21(5.1)	18(6.9)
Surface waters	271(23)	24(2.5)	24(3.8)	24(4.9)	24(5.3)
Impact	263(24)	43(1.2)	30(2.8)	27(3.7)	19(6.8)
Agriculture	262(25)	17(3.3)	20(5.0)	21(5.1)	30(3.8)
SWAT↑	261(26)	261(0.1)	180(0.6)	26(3.8)	15(8.1)
Streams↑	255(27)	50(0.9)	25(3.7)	21(5.1)	25(5.0)
Pesticides↓	186(28)	14(3.6)	21(4.8)	36(2.5)	40(2.5)
Basins↑	179(29)	110(0.5)	41(1.8)	29(3.3)	27(4.1)
watershed management	168(30)	28(1.9)	23(4.3)	28(3.4)	56(1.8)

NA: number of articles; R(%): rank and its percentage in different periods. Arrows indicate an increasing (upward arrow) or decreasing (downward arrow) trend in frequency and rank.

**Table 6:** The top 20 keywords with strong citation bursts.

Keywords	Strength	TA	Timespan and Duration					
			91–97	98–03	04–09	10–15	Begin	End
China	33.6163	141	0	0	0	141	2012	2015
Swat	16.708	188	0	0	0	188	2010	2015
groundwater	13.33	115	46	69	0	0	1995	1998
herbicide	13.0964	45	20	25	0	0	1991	2002
climate change	12.4529	80	0	0	0	80	2012	2015
geographic information system	11.8818	109	38	71	0	0	1992	2000
impact	10.5447	156	0	0	0	156	2013	2015
atrazine	10.269	56	20	17	6	13	1991	2001
erosion	9.7417	60	25	35	0	0	1992	1999
watershed management	8.9929	111	0	51	60	0	2001	2004
pesticide	8.9333	127	25	57	45	0	1996	2004
discriminant analysis	8.6377	27	0	14	13	0	2002	2007
Midwestern united states	7.7492	16	12	4	0	0	1994	2001
lake	7.4057	59	16	43	0	0	1997	2001
area	6.9597	72	0	0	0	72	2013	2015
corn	6.2069	16	10	6	0	0	1991	2001
nonpoint source	5.9653	49	49	0	0	0	1993	1994
movement	5.8849	13	9	4	0	0	1992	1999
AGNP	5.5197	32	7	19	6	0	1997	2006
galaxy	5.1373	12	8	4	0	0	1993	1998

NA: number of articles.

## Data Availability

Not applicable.
